# Theoretical Analysis of the Stationary Transport of 1:1 Salt Ions in a Cross-Section of a Desalination Channel, Taking into Account the Non-Catalytic Dissociation/Recombination Reaction of Water Molecules

**DOI:** 10.3390/membranes10110342

**Published:** 2020-11-13

**Authors:** Makhamet Urtenov, Vitaly Gudza, Natalia Chubyr, Inna Shkorkina

**Affiliations:** 1 Department of Applied Mathematics, Kuban State University, 350040 Krasnodar, Russia; flash.wetal@mail.ru (V.G.); shkorkina_inna@mail.ru (I.S.); 2 Department of Applied Mathematics, Kuban State Technological University, 350042 Krasnodar, Russia; chubyr-natalja@mail.ru

**Keywords:** ion-exchange membrane, mathematical modelling, using overlimiting current modes, membrane systems, cation-exchange membrane, effect of the breakdown of the space charge

## Abstract

In electromembrane systems, the theoretical study of salt ion transport usually uses mathematical models of salt ion transport in the depleted diffusion layer of ion-exchange membranes. This study uses a one-dimensional mathematical model of salt ion transport in a cross-section of a desalination channel formed by anion-exchange and cation-exchange membranes, taking into account an effect of a dissociation/recombination reaction of water molecules. The reaction on the one hand leads to an overlimiting mass transfer due to the effect of exaltation of the limiting current. On the other hand, an appearance of new electric charge carriers (hydrogen and hydroxyl ions) can reduce the space charge that occurs in membranes and suppress an electroconvective mechanism of overlimiting transport. Thus, there is a problem of studying these phenomena together, taking into account their mutual influence, and this article is devoted to the solution of this problem. Theoretically, using a method of mathematical modeling and numerical research, main regularities are established; in particular, it is shown that the dissociation/recombination reaction of water molecules does not lead to the destruction of the double electric layer at the membranes, but also creates a new double electric layer in the middle of the desalination channel. Thus, the space charge and the dissociation/recombination reaction significantly affect each other and simultaneously the transport of salt ions.

## 1. Introduction

Electrodialysis is widely used in wastewater treatment processes, including for toxic heavy metal salts [1,2,3,4]. Since the cost of the electrodialysis process depends on the surface area of ion-exchange membranes, the maximum possible current densities are used to increase the efficiency of the process [5,6]. At the same time, it is known that at overlimiting current densities (the limiting current is hereinafter referred to as the limiting diffusion current [7]), many different phenomena occur, the main of which are the dissociation/recombination reaction of water molecules, the appearance of an expanded space charge region, which leads to the emergence and development of electroconvection [8], the main mechanism of overlimiting transport.

Water splitting in systems with ion-exchange membranes was first observed by Frillett [9], as well as by Cressman and Tai [10], and has since been studied in articles [11,12,13,14]. Taking into account the effect of the dissociation/recombination reaction of water molecules is important for understanding the processes of ion transport in electromembrane systems, since the appearance of new charge carriers $H^{+}$ and $OH^{-}$ can lead to a decrease or even disappearance of the space charge. Currently, it is considered that there are two different mechanisms of water dissociation in electromembrane systems, namely, catalytic and non-catalytic. According to the catalytic mechanism of water dissociation on ion-exchange membranes, ions $H^{+}$ ($OH^{-}$) can occur in proton transport reactions between charged groups in the membrane and water molecules [15,16]. It was found that water dissociation is more pronounced on anion-exchange membranes (AEM) and less significant on cation-exchange membranes (CEM) [16]. In a number of works [17,18], we have shown that, in the expanded region of the space charge, the electric field intensity reaches such high values that it pulls apart the hydrogen and hydroxyl ions in this region and a non-catalytic reaction of dissociation of water molecules occurs at the maximum possible speed.

The study of all these phenomena simultaneously presents a complex problem. Therefore, the appearance of an extended space charge region and its accompanying electroconvection and the effect of the dissociation/recombination reaction of water molecules on the transport of salt ions in these articles are theoretically studied separately. At the same time, these phenomena mutually affect each other, so it becomes important to take into account their combined effect on the transport of salt ions.

In study [19], it was shown that, using gels, it is possible to achieve suppression of electroconvection, despite the presence of an expanded space charge region. At the same time, modification of the membrane surface reduces the catalytic dissociation of water molecules to almost complete disappearance [20,21]. This leaves two inextricably linked phenomena, namely, the non-catalytic dissociation/recombination reaction of water molecules and the appearance and development of an extended space charge region and their effect on salt ion transport, which must be studied together. This article is devoted to solving this problem. The article shows theoretically that the dissociation/recombination reaction of water molecules not only does not lead to the destruction of the double electric layer in ion-exchange membranes, but also creates a new double electric layer in the middle of the desalination channel both at prelimiting currents and at overlimiting currents. The main regularities of salt ion transport have been established, and it is shown that, even at prelimiting but close to the limiting currents, the effect of the dissociation reaction becomes significant, the concentration of hydrogen and hydroxyl ions becomes comparable to the concentration of sodium and chlorine ions. Thus, the space charge and the dissociation/recombination reaction of water molecules significantly affect each other and simultaneously the transport of salt ions.

## 2. Description and Analysis of Mathematical Model of One-Dimensional Non-Stationary Ion Transport in Membrane Systems

**Figure 1:**
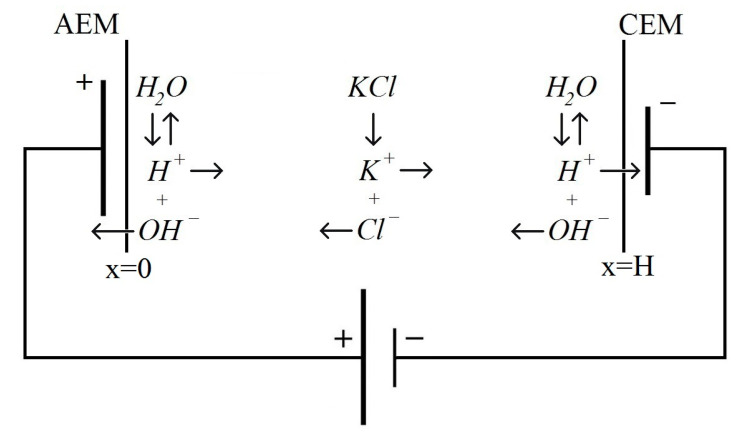
Schematic diagram of the cross-section of the desalination channel with complete dissociation of KCl and the dissociation/recombination reaction of water molecules that occur in the entire cross-section of the channel.

In this paper, we consider the cross-section of the channel desalting in the one-dimensional case, where the coordinate x changes from 0 to H, where H is the width of the channel cross-section, and the point x=0 corresponds to the border of the anion-exchange membrane (AEM)/solution, and point x=H to the boundary of the solution cation-exchange membrane (CEM), as shown in Figure 1.

Near ion-exchange membranes (see Figure 1), ions $OH^{-}$ and $H^{+}$ are transported through the anion-exchange membrane (AEM) and cation-exchange membrane (CEM), respectively. Therefore, near AEM, the solution is acidified, and CEM is alkalinized. The problem of the study is to find out what happens in the internal cross-section points of the desalination channel.

### 2.1. System of Equations

In this paper, we study four types of ions: ions $K^{+}$, $Cl^{-}$, $H^{+}$ and $OH^{-}$, which appear when water molecules dissociate. The mathematical model consists of a system of stationary one-dimensional Nernst–Planck–Poisson Equations (2) and (3) [7], and equations of dissociation/recombination of water molecules (1), (4). These equations represent the laws of conservation of material balance and electric charge. In addition, the current flow condition (5) is set, which means that the current in this system is determined by the ion flow.

Thus, the mathematical model of one-dimensional stationary transport of binary electrolyte in the cross-section of the desalination channel is described by the system of Nernst–Planck–Poisson equations:

(1) $$\frac{dj_{i}}{dx}=R_{i},\;i=1,\ldots ,4$$

(2) $$j_{i}=-z_{i}\frac{F}{RT_{0}}D_{i}C_{i}\frac{d\varphi }{dx}-D_{i}\frac{dC_{i}}{dx},\;i=1,\ldots ,4$$

(3) $$\frac{d^{2}\phi }{dx^{2}}=-\frac{F}{\varepsilon_{r}}(z_{1}C_{1}+z_{2}C_{2}+z_{3}C_{3}+z_{4}C_{4})$$

(4) $$R_{1}=R_{2}=0,\;R_{3}=R_{4}=k_{d}C_{H_{2}O}-k_{r}C_{3}C_{4}=k_{r}(k_{w}-C_{3}C_{4})$$

(5) $$I_{c}=z_{1}j_{1}+z_{2}j_{2}+z_{3}j_{3}+z_{4}j_{4}$$

$$z_{1}=1,\;z_{2}=-1,\;z_{3}=1,\;z_{4}=-1$$

Here, $\varepsilon_{r}$—the permittivity of the solution, F—the Faraday number, i=1, 2—the indices of salt ions, i=3  and  i=4—respectively, the indices of ions $H^{+}$ and hydroxyl $OH^{-}$ ions, φ—the potential jump, and $E=-\frac{d\varphi }{dx}$—the electric field strength. $C_{i}$, $j_{i}$, $D_{i}$—respectively, the concentration, flow, and diffusion coefficient of the *i*-th ion. In addition, $k_{d}=2\times 10^{-5}\;\frac{1}{s}$—the rate constant of dissociation of water molecules, $C_{H_{2}O}=55.6\times 10^{3}\;\frac{\mathrm{mol}}{m^{3}}$—the concentration of water in solution, $k_{r}=1.33\times 10^{8}\;\frac{m^{3}}{\mathrm{mol}\cdot s}$—the rate constant of recombination of hydrogen and hydroxyl ions, $k_{w}=\frac{k_{d}C_{H_{2}O}}{k_{r}}=10^{-8}\;\frac{{\mathrm{mol}}^{2}}{m^{6}}$—the equilibrium constant, and $I_{c}$—the conduction current due to the flow of ions.

### 2.2. Boundary Conditions

Equations (1)–(5) are supplemented by boundary conditions that describe the transport of ions through ion-exchange membranes. Ion-exchange membranes are considered to be perfectly selective (only counterions are passed through), and the values of counterions are determined by the exchange capacity of the membranes. Any possibility of proton leak was also disregarded. Therefore, as boundary conditions at the solution/ion-exchange membrane boundary, the impermeability conditions for salt coions (6), (12), and the values of counterions (7), (11) corresponding to the exchange capacity of ion-exchange membranes are set. A constant potential jump is also set $\Delta_{r}\varphi$: (10), (15). Since this problem considers the non-catalytic reaction of dissociation/recombination of water molecules, in conditions (8) and (14) the value is $j_{3a}=j_{4k}=0$. Conditions (9), (13) mean that the exchange capacity of ion-exchange membranes is large enough and, therefore, hydrogen and hydroxyl ions are freely transported through the cation-exchange and anion-exchange membranes, respectively.

Thus, the boundary conditions have the form (6)–(15):

(6) $${\left(-\frac{F}{RT}C_{1}D_{1}\frac{d\varphi }{dx}-D_{1}\frac{dC_{1}}{dx}\right)|}_{x=0}=0$$

(7) $$C_{2}(0)=C_{2a}\;$$

(8) $${\left(-\frac{F}{RT}C_{3}D_{3}\frac{d\varphi }{dx}-D_{3}\frac{dC_{3}}{dx}\right)|}_{x=0}=j_{3a}$$

(9) $$\frac{dC_{4}(0)}{dx}=0$$

(10) $$\varphi (0)=\Delta_{r}\varphi$$

(11) $$C_{1}(H)=C_{1k}\;$$

(12) $${\left(\frac{F}{RT}C_{2}D_{2}\frac{d\varphi }{dx}-D_{2}\frac{dC_{2}}{dx}\right)|}_{x=H}=0$$

(13) $$\frac{dC_{3}(0)}{dx}=0\;$$

(14) $${\left(\frac{F}{RT}C_{4}D_{4}\frac{d\varphi }{dx}-D_{4}\frac{dC_{4}}{dx}\right)|}_{x=H}=j_{4k}$$

(15) φ(H)=0

## 3. Results

### 3.1. Analysis of the Boundary Value Problem without Taking into Account the Dissociation/Recombination Reaction

It is easy to show that, in the absence of a dissociation/recombination reaction of water molecules, the boundary value problem (1–4) with the corresponding boundary conditions has a solution only for zero current. Indeed, we put in the system of equations $C_{3}(x)=C_{4}(x)\equiv 0,\;j_{3}(x)=j_{4}(x)\equiv 0$. Then, to find the concentrations and fluxes of salt ions, we obtain the boundary value problem:

(16) $$\frac{dj_{i}}{dx}=0,\;i=1,2$$

(17) $$j_{i}=-z_{i}\frac{F}{RT_{0}}D_{i}C_{i}\frac{d\varphi }{dx}-D_{i}\frac{dC_{i}}{dx},\;z_{1}=1,z_{2}=-1,\;i=1,2$$

(18) $$\frac{d^{2}\phi }{dx^{2}}=-\frac{F}{\varepsilon_{r}}(C_{1}-C_{2})$$

From (5), it follows that $j_{1}=const$ and $j_{2}=const$, and, from the boundary conditions (6) and (12), we obtain:

$$j_{1}={\left(-\frac{F}{RT}C_{1}D_{1}\frac{d\varphi }{dx}-D_{1}\frac{dC_{1}}{dx}\right)|}_{x=0}=0$$

$${j_{2}=\left(\frac{F}{RT}C_{2}D_{2}\frac{\partial \varphi }{\partial x}-D_{2}\frac{\partial C_{2}}{\partial x}\right)|}_{x=H}=0$$

**Figure 2:**
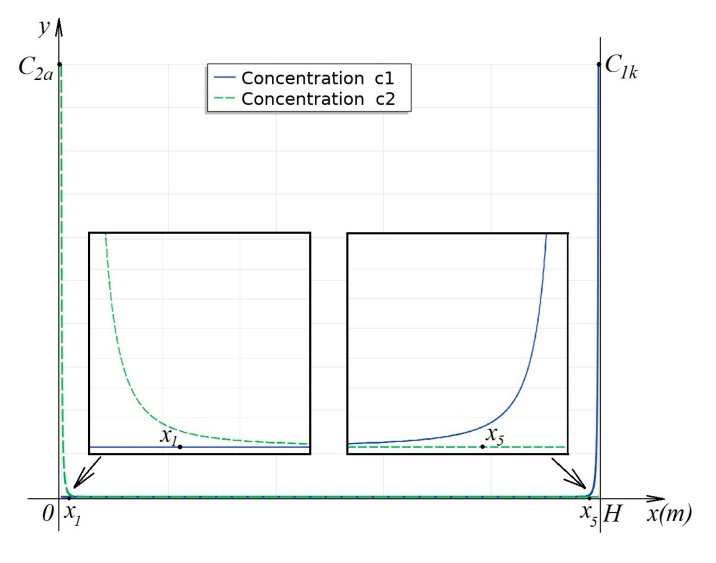
Distribution of concentration profiles $K^{+}$ and $Cl^{-}$ in the channel cross – section for the stationary case at $j_{1}=j_{2}\equiv 0$: $x_{1}$ and $x_{5}$—boundaries of quasi-equilibrium space charge regions near anion-exchange membranes (AEM) and cation-exchange membranes (CEM), respectively. Between points $x_{1}$ and $x_{5}$, the concentration value is small.

Thus,$j_{1}=j_{2}\equiv 0$, hence $I_{c}=j_{1}-j_{2}\equiv 0$. The obtained problem is classical for the diffusion layer and its solution is well known [7]. Its generalization to the case of a channel cross-section is easy, in accordance with Figure 2:

As will be seen below, taking into account the dissociation/recombination reaction changes the solution qualitatively. Since the relation $j_{1}=j_{2}\equiv 0$ is also fulfilled in the main problem (1–15), the conduction current $I_{c}$ is completely determined by the products of dissociation of water molecules.

### 3.2. Results of Numerical Calculations

For numerical calculations, the finite element method is used in the mathematical modeling environment COMSOL Multiphysics 5.5. The calculations used standard values of well-known model parameters and, in addition, H=1  mm, the potential jump Δφ varies in numerical experiments from 0.001 V to 2.50 V, $C_{2a}=C_{1k}=0.1\;{\mathrm{mol}/m}^{3}$.

To analyze the transport process, the following functions are used along with the distribution of concentrations, intensity, and potential of the electric field:

(1) Equilibrium function for the dissociation/recombination reaction of water molecules: $f_{w}(C_{3}(x),C_{4}(x))=k_{w}-C_{3}(x)C_{4}(x)$. At the points where $f_{w}(C_{3}(x),C_{4}(x))>0$, the dissociation reaction dominates the recombination, and in $f_{w}(C_{3}(x),C_{4}(x))<0$—dominated by recombination.
(2) Charge density distribution: $\rho (x)=F\cdot (C_{1}(x)-C_{2}(x)+C_{3}(x)-C_{4}(x))$.

Numerical calculations show that when the potential jumps are smaller than 0.1 V, the effect of dissociation is almost imperceptible, since the concentrations of hydrogen and hydroxyl ions are hundreds of times lower. Starting with the potential jump 0.3 V, the concentration of hydroxyl ions and salt ions becomes of the same order and, therefore, it is necessary to take into account the dissociation/recombination reaction, although this potential jump corresponds to a prelimiting current.

**Figure 3:**
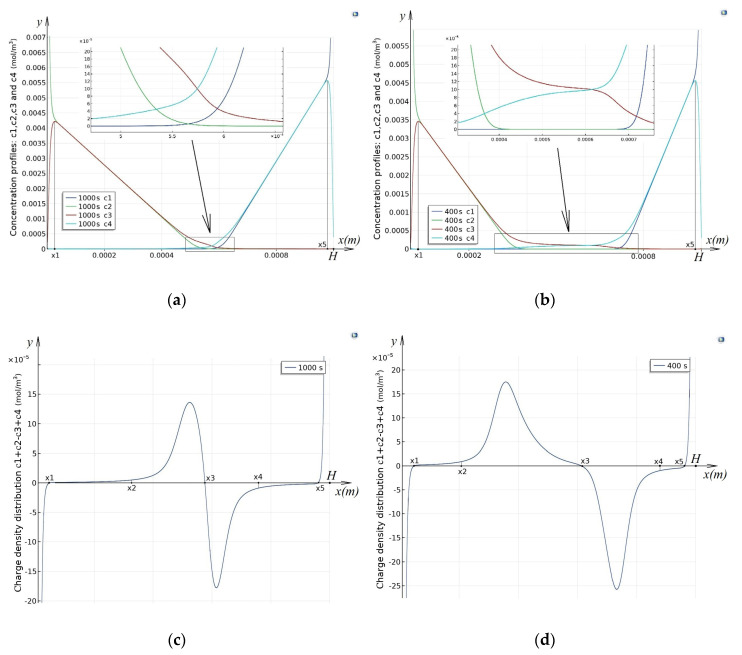

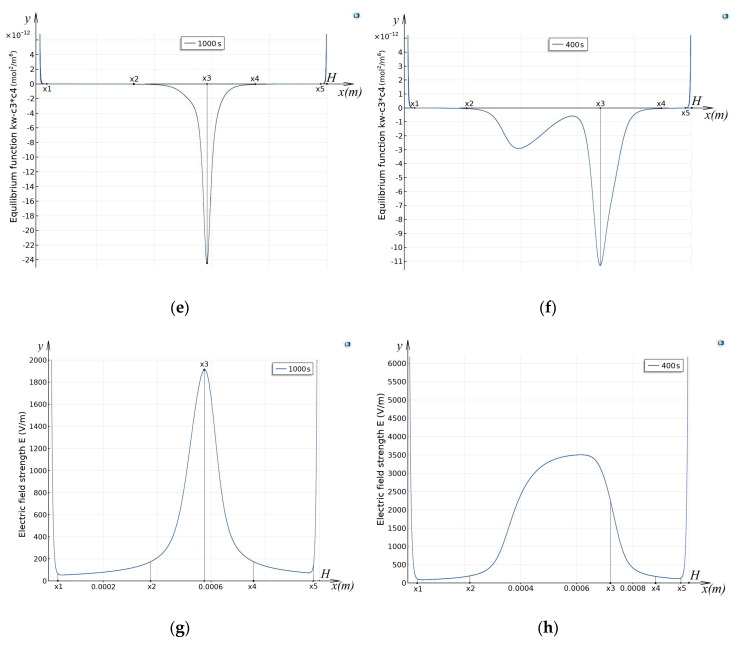
Graphs of concentration functions (**a**,**b**), charge distribution density (**c**,**d**), equilibrium (**e**,**f**), electric field strength (**g**,**h**). The left column corresponds to $\Delta_{r}\phi =0.5\;V$, and the right—$\Delta_{r}\phi =1.5\;V$. Points, $x_{1},\;\ldots ,\;x_{5}$ define the boundaries of regions (see in Section 3.3, Section 3.4 and Section 3.5).

In the future, we will analyze the transport process for two different potential jumps: $\Delta_{r}\phi =0.5\;V$ when the anion-exchange membrane has a prelimiting mode, and the cation-exchange membrane has an overlimiting mode, and $\Delta_{r}\phi =1.5\;V$ when the overlimiting mode is set for both membranes, in accordance with Figure 3:

The analysis of Figure 3 shows that the cross-section of the desalination channel can be divided into several regions.

### 3.3. Quasi-Equilibrium Regions of Space Charge

Two regions adjacent to the ion-exchange membranes, namely, $\left[0,\;x_{1}\right)$ and $\left(x_{5},\;H\right]$, are the regions of space charge (Figure 3c,d). In these regions, the electric field strength takes on very large values (Figure 3g,h), which leads to intense dissociation of water molecules (Figure 3e,f). In region $\left[0,\;x_{1}\right)$ $OH^{-}$ ions are completely transported through the anion-exchange membrane (see boundary condition 12), so the concentration of these ions is almost zero. At the same time, the concentration of ions $H^{+}$ is growing rapidly. $Cl^{-}$ ion concentration drops exponentially from $C_{2a}$, and the concentration $K^{+}$ is almost zero. Similarly, with appropriate refinements, there is a transport of ions in the region $\left(x_{5},\;H\right]$. Comparing Figure 3a–d, and so on, we can conclude that all the characteristics of these regions practically do not depend on the potential jump, so they are quasi-equilibrium regions of space charge.

### 3.4. Region of Recombination

In the region $\left(x_{2},\;x_{4}\right)$, according to Figure 3e,f, recombination dominates dissociation, so we will call it the recombination region. When the potential jump is $\Delta_{r}\phi =0.5\;V$, the structure of the recombination region is almost symmetrical relative to the middle of the channel, with a slight shift to the right, since the diffusion coefficients of potassium and chlorine ions are almost the same. A characteristic feature is the presence of a deep single local minimum of the equilibrium function due to the dominance of recombination over dissociation (Figure 3e) The minimum point is determined by the fact that, at this point, the concentration of hydrogen ions is equal to the concentration of hydroxyl ions (Figure 3a) and that is why recombination occurs at this point as much as possible. To the left of the minimum point, the concentration of hydrogen ions is greater than the concentration of hydroxyl ions. Since the concentration of potassium and chlorine ions in the recombination region is significantly lower than the concentration of hydrogen and hydroxyl ions (Figure 3a), an excess of hydrogen ions is formed to the left of the minimum point, i.e., a region of positive space charge appears (Figure 3c). Similarly, to the right of the minimum point, there is a region of negative space charge formed by an excess of hydroxyl ions.

When the potential jumps are $\Delta_{r}\phi =1.5\;V$, the difference in the diffusion coefficients of potassium and chlorine ions affects much more. The space charge region is more extended, although the maximum and minimum values do not differ much (Figure 3d). A special feature is also the presence of two local minimum of the equilibrium function (Figure 3f), which is due to the fact that the electric field strength between these points is very high. This leads to the fact that the dissociation increases so much that almost an equilibrium is reached at the point of the local maximum of the equilibrium function, which is located between two local minimums.

### 3.5. Regions of Electroneutrality and Equilibrium

In the regions $\left(x_{1},\;x_{2}\right)$ and $\left(x_{4},\;x_{5}\right)$, according to Figure 3c–f, the condition of local electroneutrality and equilibrium is met (therefore, the fluxes of hydrogen and hydroxyl ions are constant). In these regions, the distribution of concentrations is almost linear, in region $\left(x_{1},\;x_{2}\right)$, local electroneutrality is being performed due to the equality of concentrations of chlorine and hydrogen ions $C_{2}(x)=C_{3}(x)=-\frac{j_{3}}{2D_{3}}x+C_{2}(x_{1})$, and, in region $\left(x_{4},\;x_{5}\right)$, local electroneutrality is being performed due to the equality of concentrations of potassium and hydroxyl ions.

## 4. Conclusions

In this paper, the stationary transport of binary salt ions in the cross-section of the desalination channel is analyzed theoretically for the first time using a mathematical model. The main regularities are established, namely, it is shown that the cross-section of the desalination channel consists of narrow quasi-equilibrium regions of space charge adjacent to ion-exchange membranes; in the central part, there is a fairly extensive recombination region, where recombination dominates dissociation. It is shown for the first time that a double electric layer of hydrogen and hydroxyl ions appears in the recombination region. It is shown that, between the recombination region and the quasi-equilibrium regions of the space charge, there are regions of electroneutrality and equilibrium with an almost linear distribution of concentrations. It has been found that, under prelimiting conditions, but rather close to the limiting current, non-catalytic dissociation of water molecules in the quasi-equilibrium region of the space charge occurs so intensively that the concentration of hydrogen and hydroxyl ions becomes comparable to the concentration of potassium and chlorine ions. Thus, the space charge and the dissociation/recombination reaction of water molecules significantly affect each other and simultaneously the transport of salt ions.
